# Executive Function Training Improves Emotional Competence for Preschool Children: The Roles of Inhibition Control and Working Memory

**DOI:** 10.3389/fpsyg.2020.00347

**Published:** 2020-03-10

**Authors:** Quan Li, Peiwei Liu, Ni Yan, Tingyong Feng

**Affiliations:** ^1^Collage of Teacher Education, Qujing Normal University, Qujing, China; ^2^Faculty of Psychology, Southwest University, Chongqing, China; ^3^Department of Psychology, University of Florida, Gainesville, FL, United States

**Keywords:** executive function, executive function training, 2 months, emotional competence, preschool children

## Abstract

The study examined how executive function (EF) training could improve children’s emotional competence (EC). Children (*N* = 55; *M*_age_ = 50.64 months) were assigned into two groups, namely the EF training group and the no-training group. The present study attempted to use a 2 (group: EF training VS no-training) × 2 (test time: pretest VS post-test) between-and- within-subjects experimental design to investigate the effect of EF training on the improvement of EC for 4-year-old children. Results showed that, (1) children in EF training group had significantly higher scores on EC than that of no-training group; (2) The change of inhibition control and working memory could significantly predict their variation of EC. These results suggested that the improvement of EC caused by EF training could be linked to the ability of inhibition control and working memory.

## Introduction

Emotional competence (EC) refers to the abilities of recognize, comprehend, express, and regulate emotions (Mirabile, 2009; Lahaye et al., 2011; Riquelme and Montero, 2013). Emotional competence is developed throughout childhood (Liu and Chen, 2003; Pons et al., 2003). It is a fundamental ability that can help children to handle a challenge, establish a social relationship, keep mental health, and adapt to various social environment (Wang et al., 2014; Wardiesielski et al., 2019). It has been shown that children’s emotional competence is associated with their executive function (EF) (Rhoades et al., 2009; Smith et al., 2014; Kwok et al., 2015; Lantrip et al., 2015; Healy et al., 2018). However, the causal relation between them is unclear due to the limited evidence from previous experimental studies. The study aimed to determine the extent to which EF training could improve children’s emotional competence.

Executive function involves four basic abilities including inhibition control, cognitive flexibility, working memory, and problem-solving (Miyake et al., 2000; Hong et al., 2004; Oh and Lewis, 2008; Weyandt et al., 2014). Children with better emotional competence tend to exhibit better ability of inhibition control, shifting and problem-solving, but less impulsivity (Zelazo et al., 2003, 2010; Silkenbeumer et al., 2016).

Previous studies found that children’s emotional competence involved affective system, attentional system and self-control system (Zhou and Zhou, 2003; Zelazo and Carlson, 2012). Silkenbeumer et al. (2016) found that children’s development of emotional competence required the ability of internalization inhibition and modified the elicited emotional action. Moreover, inhibition control can influence children’s emotional competence. Gerstadt et al. (1994) used Day/Night task to assess children’s EF (e.g., Inhibition control ability), and Kusche (1984) used Emotional Inventory task to measure children’s emotional competence ability (Kusche, 1984; Gerstadt et al., 1994). The study found that better ability of inhibition control had better performance of emotional competence (Fox et al., 2001; Fox and Calkins, 2003; Liew, 2012). Rhoades et al. (2009) demonstrated that children who had better EF were more likely to be higher on social-emotional skills and lower on problem behaviors in a sample of 146 preschools. Likewise, Garcia-Andres et al. (2010) investigated the relationship between EF and emotional regulation in 7 and 8-year-old children. They gave children Wisconsin Card Sorting Test (WSCN), Backward Digit Span (DIG), Five Digit Test (Stroop effect), and Tower of Hanoi (ToH) to measure their EFs. They found that children with the better EF performed better in the use of emotional regulation strategies (Garcia-Andres et al., 2010). Notably, children’s EF (e.g., inhibition control) can promote the development of emotional strategies.

Executive function is related to individual emotional competence, but to date, it is unclear to the causal relationship between them. Previous literature has shown that children’s EFs could influence children’s ECs. However, to our knowledge, there is no strong causal evidence to demonstrate whether EF training can influence emotional competence. We state that children’s emotional competence needs EF to better adjust to the present situation. For instance, the test of emotional comprehension includes the understanding of belief-based emotions, and it is well-documented that belief-based reasoning involves EF. Moreover, the test of emotional regulation and emotional expression might require inhibition control. For example, the children should control their own negative emotion (e.g., anger) during interaction to better manage emotions. To address this issue, we used a 2 (groups: No-training VS EF training) × 2 (test time: pretest VS post-test) between-and-within-subjects experimental design (that is experimental and control group pretest–posttest design). In EF training group, the well-trained research assistant guided children to carry out EF training activities (20∼30 min every time, twice a week, and 12 times in total). In No-training group, the children participated in daily class activities without EF training at the same time. Two hypotheses were examined: (1) EF training group would have a greater increase in emotional competence than no-training group after the intervention, (2) Children’s EF change would explain the improvement of children’s emotional competence.

## Method

### Participants

Fifty-five healthy Chinese preschoolers (EF training group: *N* = 29, *M*_age_ = 50.65 months, *SD* = 3.11 months; No-training group, *N* = 26, *M*_age_ = 50.63 months, *SD* = 3.32 months) participated in the study. These children were recruited from two classes (middle classes, age = 4∼5 years old) of local kindergartens. Four children of No-training group Were excluded from the analyses because they didn’t participate in the post-test. The intervention took place over a 2-month period and involved fifty-one children. We Have modified them as “six children of EF training group participated EF training at One time. All children were given informed consent for this study (parental consent and participant assent for children). The study was approved by the Southwest University’s Academic Ethics Committee (IRB: H19063).

### Design

The study used a 2 (group: No-training VS EF training) × 2 (test time: pre VS post) between- and-within-subjects experimental design. The group (group: No-training VS EF training) was the between-subject variable but the test time (test time: pre VS post) was the within-subject variable. The No-training group was control group, and the EF training group was experimental group. Otherwise, the study was approved by the Southwest University’s Academic Ethics Committee.

### Measures and Materials

According to previous literatures, Facial Expression Match and Recognition, Test of Emotion Comprehension (TEC), Situational Storytelling of Emotion Expression, and Situational Storytelling of Emotion Regulation were widely used to measure the ability of children’s emotional competence capacity (Dodge, 1989; Carlson et al., 2002; Pons et al., 2002; Ling and Ya, 2003; He et al., 2005; Bierman et al., 2008; Mirabile, 2009; Lahaye et al., 2011; Dong, 2012; Riquelme and Montero, 2013). The Emotional Stroop Test, Dimensional Change Card Sorting (DCCS), Memory for Picture of Wechsler Intelligence Scale and Situational Storytelling of Problem Solving was used to measure children’s EF (Wechsler, 1974, 2003; Ruff et al., 1998; Miyake et al., 2000; Ruff and Capozzoli, 2003; Chrysikou and Weisberg, 2005; Zelazo, 2006; Besnier et al., 2008).

### Emotional Recognition: Facial Expression Match and Recognition

The Facial Expression Match and Recognition was used to measure children’s emotional recognition in this study (Bierman et al., 2008; Dong, 2012). All pictures in the task were selected from database of Nimstim Facial Expressions of Emotion (Tottenham et al., 2009). All pictures in the task were selected Chinese faces, and the picture numbers of male faces and female faces were equal. Children were asked to recognize pictures with four types of facial emotions (happy, sad, angry, and fear) and then match them with emotional words (e.g., which picture is happy?). Two points were assigned for each correct-recognization picture and one point for each correct-match picture, and thus the final score of emotional recognition should range from 0 to 12 points (Markham and Wang, 1996; Mo and Su, 2004; Quan et al., 2019).

### Emotional Comprehension: Test of Emotion Comprehension (TEC)

Test of Emotion Comprehension (TEC), including four sections, was used to measure children’s emotional Comprehension in this study (i.e., Belief, Desire, Reminder, and Cause) (Pons et al., 2002). The TEC test depended on a comic book with a simple cartoon scenario on each page (the size of comic book = 21 cm by 29.7 cm). There were four emotional outcomes (e.g., happy, sad, angry, and fear) on the bottom of the page. All the facial expressions typically represented each scenario. The test procedure included two steps: (1) The experimenter read the cartoon (two stories in each section). (2) The child was asked to point out the most appropriate picture of match the four possible emotional outcomes. Four sections in total with a fixed order, (I) Understanding desire-based emotions (e.g., the same situation but opposite desires individually); (II) Understanding belief-based emotions (e.g., attribution of an emotion to a chick who enjoying worms without knowing that an eagle was hiding in the tree); (III) Understanding the cue of a reminder about a present emotional state (e.g., perception of the emotion to a character that was reminded of the loss of a pet); (IV) Understanding external causes of emotions (e.g., attribution of an emotion to a character being displeased by a puck). Children would get one point when he or she answered a theme correctly. The final score of emotional comprehension was calculated by the summation of points in each.

### Emotional Expression: Situational Storytelling of Emotion Expression

The Situational Storytelling of Emotion Expression was administered to assess emotional expression (Carlson et al., 2002; He et al., 2005). Children were presented with a comic book with a simple cartoon scenario in the center (the size of comic book = 21 cm by 29.7 cm). Four situational storytelling (two positive situations and two negative situations) was included. Every situation had three questions needed to be answered by pointing to facial pictures (angry, sad, calm, and happy). For example, the experimenter read a positive situation (e.g., “Today is Mike’s birthday, Mike’s friend bought a birthday gift to him, Mike wanted to get a toy car, but when he opened the gift and found an ugly doll there. If you were Mike, what would you choose?”). Question one is about true emotion (sad or angry): “what is Mike feeling?” Question two is about ego-oriented motivation: “If Mike was shown the true emotion (sad or angry), and his friend would never send him the birthday gift. What do you think Mike should show on his face?” Question three is about social goal orientation: “If Mike showed the true emotion (sad or angry), and his friend would be very sad. What do you think Mike should show on his face?” There were four emotional expression strategies: (1) calming: when true emotion was angry, sad, or happy, but preschoolers chose calm facial picture; (2) hiding: when true emotion was sad or angry, but children chose happy facial picture; (3) exaggerating: when true emotion was sad, but preschoolers chose angry facial picture; (4) weakening: when true emotion was angry, but kids chose sad facial picture. When the strategy was appropriate for the situation. The participants would get two points for appropriate match between strategy and situation, one point when the strategy did not coincide the situation, and zero point when the children didn’t use strategy. The final score of Situational Storytelling of Emotion Expression was calculated by adding up the scores from four blocks.

### Emotion Regulation: Situational Storytelling of Emotion Regulation

The Situational Storytelling of Emotion Regulation was chosen in order to assess emotional regulation (Dodge, 1989; Ling and Ya, 2003; Dong, 2012). The Situational Storytelling of Emotion Regulation was showed by a comic book with a simple cartoon scenario (the size of comic book = 21 cm by 29.7 cm). The test of emotional regulation includes four situational storytelling (two positive situations and two negative situations). Every emotional situation had four strategies to choose after the experimenter read the situational story to the child. For instance, the experimenter read a negative situation (e.g., Mike was stacking toy blocks carefully, but his good friend walked to him and said that your toy blocks stacked bad, and his friend pushed the blocks down with saying that “let me help you to stack”) and asked the child, “what would you do if you were the main character?” Four options were provided: (1) Self-repression: No speaking, playing the other toys; (2) relying on the adult: Cry to the teacher; (3) Impulsive behavior: Pushing companion down; and (4) Self-assertion: Asking his friend to stack together. Two points would be got for choosing “Self-assertion,” one point for “Self-repression” and relying on the adult, one point for “Self-assertion” and relying on adult, but zero point for “Impulsive behavior.” The final score of Situational Storytelling of Emotion Regulation was calculated by adding up two positive and negative mean scores.

### Inhibition Control: Emotional Stroop Test

The Emotional Stroop Test was administered to children’s inhibitory control, which was an element of children’s EF (Besnier et al., 2008). An emotional picture was used as an automatic process in the Emotional Stroop Test. Participants was asked to ignore the word meaning of words when they were instructed to read the facial expression (Bentall and Kaney, 1989; Cothran and Larsen, 2008). The Emotional Stroop Test required participants to inhibit the word on the facial expression pictures, but answered to facial expression ignoring the meaning of word. The timed reaction and accuracy were analyzed during the Stroop task. In our study, eight trials were presented to children as the exercise for understanding experimental rules. The experimenter told children “When the happy facial expression is shown on the screen, you tell me “unhappy.” The final experiment included three sessions. Every session presented 16 trails with 8 happy faces and 8 unhappy faces with the random order. All pictures in the task were selected from database of Nimstim Facial Expressions of Emotion (Tottenham et al., 2009). Considering that children in the study were too young to make choice by pushing the button (aged from 46 to 56 months), the experimenter helped them push it. Given that, we only recorded accurate rate but ignored reaction time.

### Cognitive Flexibility: Dimensional Change Card Sorting

We selected Dimensional Change Card Sorting (DCCS) with the version of 3∼5 years-old children (Gerstadt et al., 1994; Zelazo et al., 1996; Zelazo, 2006) to assess the cognitive flexibility in our study. Firstly, the dimensions had two parts (color dimension and shape dimension), which was relevant during the pre-switch phase as the standard version. For instance, if the color dimension had been chosen as a pre-switch dimension, and the shape was a post-switch dimension. Practicing block included six switch trails (three color switch trails and three shape trails). Secondly, the experimenter told the children, “Now we’re going to play a color-shape game. In the color game, all green ones gone here [pointing to the green tray], and all white ones gone here [pointing to the white tray]. In the shape game, now we’re going to play a new game, all rabbits gone here [pointing to the rabbit tray], and all boats gone here [pointing to the boat tray].” Children aged from 3 to 5 years old usually sorted correctly on all six pre-switch color trials. The child who could sort five out of six post-switch trials correctly was regarded as the pass for the post-switch. A score of 0 was assigned if the child could not pass the pre-switch phase of standard version; a score of 1 was assigned if the child passed the pre-switch phase of standard version but failed the post-switch phase; 2 points if the child passed both pre-switch and post-switch of standard version but failed the next switch. The final of score of Dimensional Change Card Sorting (DCCS) ranged from 0 point to 6 points.

### Working Memory: Memory for Picture of Wechsler Intelligence Scale for Children (IV)

Memory for Picture of Wechsler Intelligence Scale for Children (IV) was administered in order to measure preschool children’s working memory, which was an essential component of EF (Wechsler, 1974, 2003). In the present study, we chose the normative testing for 4 year-old children. The test of working memory included 35 sets of pictures, which ranged from one picture to six pictures in each set. Children was asked to point out the target pictures that the experimenter showed. One point was given when each question was correctly answered, and the test was ended when the child failed to answer four sets of questions continuously. The final score of children’s working memory was calculated by the sum up all sets together.

### Problem Solving: Situational Storytelling of Problem Solving

The Situational Storytelling of Problem Solving was administered in order to measure Problem Solving as a part of EF (Ruff et al., 1998; Ruff and Capozzoli, 2003; Chrysikou and Weisberg, 2005). The Situational Storytelling of Problem Solving relied on a comic book with a simple cartoon scenario on the page (the size of comic book = 21 cm by 29.7 cm), which included two situational storytelling (one positive situation and one negative situation). Every situation had four strategies to choose after the experimenter read the situational story. For instance, the experimenter read a positive situation (e.g., Mike and Lily play slide together after reciting children’s song, Mike had recited, but Lily couldn’t recite), and the experimenter asked the child, “what would you do if you were Mike to play slide with Lily as soon as possible?” The children would get two points if he or she choose “A: I would help her to recite”; he would get one point if he choose “B: Waiting for her to recite”; he would get half of a point when he choose “C: I didn’t wait for him to play slide by myself,” and he would get zero points when he choose “D: I don’t know.” The final score of Situational Storytelling of problem solving was the mean of the points of two situations (Dennis et al., 2009; Suor et al., 2017).

#### Procedure

After getting the consent from the parents and kindergarten’s permission, children were tested individually in a quiet room. All the tests were divided into two sections. Each session lasted approximately 35–40 min. As a warm-up, the experimenters asked the child for whether he or she liked to play games and which games he or she played usually. The experimenter measured the children’s EFs and ECs before and after. Of note, the Facial Expression Match and Recognition was assessed at the start of pretest and post-test, and other measures were counterbalanced across participants.

### Manipulation

Children from two different kindergarten classes (Class 1: 29 children; Class 2: 26 children) were randomly assigned to EF training group (*N* = 29) and No-training group (*N* = 26).

#### Training Group

Twenty-nine children were randomly assigned to the EF training group. The self-developed EF training curriculum was based on four EF sub- components, which was used for the age of 4–5 year-old children. The training sessions was twice a week over 2 months (12 session in total). In order to have a better training effect, only 5 or 6 children took the 20–30-min EF training class for each time by well-trained research assistants: (1) 1^st^–4^th^ session, the aim is to promote the inhibition control ability; (2) 5^th^–6^th^ session, the aim is changed to promote the cognitive flexibility; (3) 7^th^–8^th^ session, the aim is to develop the working memory; (4) 9^th^–10^th^ session, the aim is to improve the problem solving ability; (5) 11^th^–12^th^ session, the aim is to review all the training classes above. (Detailed curriculums see Table 1).

**TABLE 1:** The syllabus of EF training class.

**Training abilities**	**Sessions**	**Curricular title**	**Example items**
Inhibition control	1–4	Big and small watermelon	**Aim:** Improve the conflict control ability. **Operation:** When research assistant gesticulate “big watermelon,” children should answer “small watermelon.” When research assistant gesticulate “small watermelon,” children should answer “big watermelon.” **Conflict condition:** Gig VS Small.
		Sun and star	**Aim:** Improve the conflict control ability. **Operation:** When research assistant show “sun picture,” children should answer “night.” When research assistant show “star picture,” children should answer “day.” **Conflict condition:** Day VS Night.
		Black and white magic wand	**Aim:** Improve the conflict control ability. **Operation:** When research assistant gesticulate “white,” children should answer “black.” When research assistant gesticulate “black,” children should answer “white.” **Conflict condition:** Black VS White.
		The pony across the river	**Aim:** Improve the conflict control ability. **Operation:** When research assistant show “white river,” children should walk “black river.” When research assistant show “black river,” children should walk “white river.” **Conflict condition:** Black VS White.
Cognitive flexibility	5–6	Mike’s birthday	**Aim:** Improve the cognitive flexibility. **Operation:** Shown six positive emotional scenes and six negative emotional scenes (e.g., getting birthday gift). **Shifted condition:** From positive emotion to negative emotion.
		Book was trampled	**Aim:** Improve the cognitive flexibility. **Operation:** Shown six negative emotional scenes and six positive emotional scenes (e.g., the book was trampled). **Shifted condition:** From negative emotion to positive emotion.
Working memory	7–8	Fruit platter	**Aim:** Improve the working memory. **Operation:** Reciting fruits on forward and backward [range: 2∼5] (e.g., recited forward “apple\pear\banana” and recited backward “banana\pear\apple”). **Processing sequence:** First reciting forward and backward.
		Digital overturning	**Aim:** Improve the working memory. **Operation:** Reciting digits on forward and backward [range: 2∼5] (e.g., recited forward “1\5\2” and recited backward “2\5\1”) **Processing sequence:** First reciting forward and backward
Problem solving	9–10	Lamb was eaten	**Aim:** Improve the problem solving. **Operation:** (1) Acting the story; (2) Asked the children“if you were the master, what would you do?” (e.g., Milk’s lamb was eaten by a wolf. If you were Milk, what would you do?). **Training core abilities:** problem solving\inhibition control
		Flowers was crushed	**Aim:** Improve the problem solving. **Operation:** (1) Acting the story; (2) Asked the children“if you were the master, what would you do?” (e.g., Milk’s flowers were crushed by a dog. If you were Milk, what would you do?) **Training core abilities:** problem solving\inhibition control
Combined training	11–12	Kitchen parade	**Aim:** Reconsolidating. **Operation:** (1) Divided into two groups, each group had 3 children; (2) One group cooked, the other group waited; (3) Limited ingredients, asked other children for help to cook each other; (4) Swapped position. **Training core abilities:** Combined multiple capacities.
		Painting emotional pictures	**Aim:** End the class **Operation:** (1) Shown four emotional pictures; (2) Asked children paint pictures, but different colors; (3) Limited colors, asked other children for help to paint each other. **Training core abilities:** Combined multiple capacities.

#### No-Training Group

Children in the No-training group took the normal course activities. All is same to training group but there is no EF training classes.

## Results

Firstly, in order to make the EF and EC comparable, the value of them were converted into Z scores. In the study, we got the score of EC by calculating the summation of score of emotional recognition, emotional comprehension, emotional expression, and emotional regulation. Likewise, the score of EF was calculated by summing up the scores of the inhibition control, cognitive flexibility, working memory, and problem solving.

The G^∗^Power 3 was used to compute the statistical power in this study (*N* = 55). The test effects setting was effects in within-between-subjects designs for two groups. The results showed that the power (1-β err prob) was 0.997, suggesting a strong statistical power in our study (Faul et al., 2007).

### Pretest Data Analysis

To corroborate earlier findings on the relationship between EF and emotional competence (EC), we performed correlation analysis between EF and EC during pretest. The result showed that it was a significantly positive association between EF and EC, *r* = 0.679, *p* < 0.001. The finding suggested that children’s ECs could be improved by training EFs.

To examine the difference of ECs between the EF training group and No-training group during pretest, we performed independent sample *t*-tests. The result showed that the EC and sub-capacities had no significant difference, *ps* > 0.05. Overall, the result revealed that EF and EC didn’t show significant difference during pretest, but No-training group performed better than EF training group on inhibition control, *t* = 2.996, *p* < 0.01. To control the impact of inhibition control during pretest, we used children’s inhibition control change to make further analysis during post-test.

### Pre–Post-test Data Analysis

The analysis revealed that the increase in the EF training group was higher than that of no training group 2 months later (see Table 2).

**TABLE 2:** Description statistics between pretest and post-test.

**Variable**	**No training**		**EF training**	
	**Pretest**	**Post-test**	**Pretest**	**Post-test**
EC	2.30	2.57	2.10	3.08
E-Reco	6.32	7.84	6.04	10.63
E-Com	2.58	2.63	2.39	2.97
E-Exp	3.97	3.45	3.63	4.14
ER	1.86	2.73	1.52	3.04

EF, executive function; EC, emotional competence; IC, inhibition control; Flex, cognitive flexibility; WM, working memory; PS, problem solving; E-Reco, emotional recognition; E-Com, emotional comprehension; E-Exp, emotional expression; ER, emotional regulation. *p < 0.05; **p < 0.01; ***p < 0.001.

### Repeated Measures

To investigate whether EF training could enhance children’s EC, we performed 2 (group: No-training VS EF training) × 2 (test time: pre VS post) repeated measures ANOVA. The emotional competence was the dependent variable. Results revealed that test time main effect was not significant, *F*(1,49) = 0.547, *p* > 0.05; the group main effect was not significant, *F*(1,49) = 2.066, *p* > 0.05; but the interaction between group and test time was significant, *F*(2,49) = 21.10, *p* < 0.001, η2 = 0.373.

**FIGURE 1:**
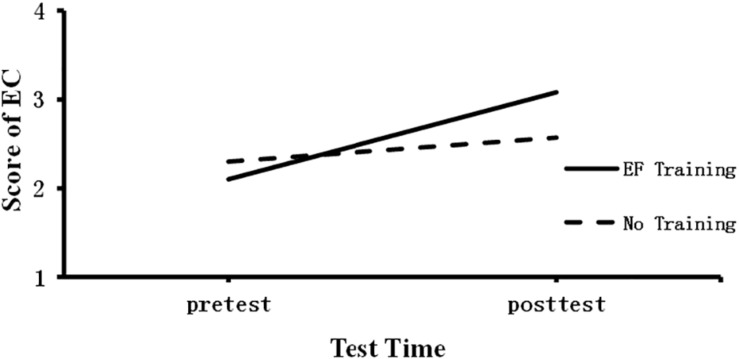
Children’s Score of EC during pre–posttest.

Further simple effect analyses showed that EF training group and No-training group were not significant during the pretest, *M*_diff_ (I–J) = 0.20, *p* > 0.05. But EF training group children performed better than No-training group during post-test, *M*_diff_(I–J) = 0.51, *p* < 0.001, η2 = 0.272 (see Figure 1).

The findings suggested that EF training can improve children’s emotional competence.

To illustrate the effect of children’s EF on the sub-capacities of children’s emotional competence.

**FIGURE 2:**
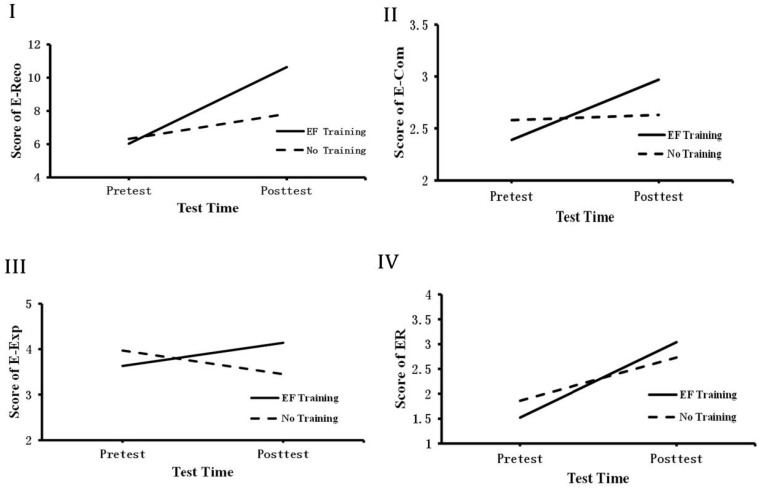
Group by test time interaction on the EC. **(I)** The scores of emotional recognition during pre–posttest; **(II)** the scores of emotional comprehension during pre–posttest; **(III)** the scores of emotional expression during pre–posttest; **(IV)** the scores of emotional regulation during pre–posttest.

First, in order to investigate whether training EF improves children’s emotional recognition, we performed 2 (group: No-training VS EF training) × 2 (test time: pre VS post) repeated measures ANOVA. The emotional recognition was the dependent variable. The results revealed test time main effect was not significant, *F*(1,49) = 0.291, *p* > 0.05; the emotional recognition ability of training group was significantly greater than that of No-training group, *F*(1,49) = 5.896, *p* < 0.01, η2 = 0.144, and the interaction between group and test time was significant, *F*(2,49) = 10.159, *p* < 0.001, η2 = 0.222. Further simple effect analyses showed that EF training group and No-training group were not significant during the pretest, *M*_diff_(I–J) = 0.086, *p* > 0.05, but EF training group children performed better than No-training group after the intervention, *M*_diff_(I–J) = 1.111, *p* < 0.001, η2 = 0.343 (see Figure 2-I). The findings suggested that EF training can improve children’s emotional recognition. Secondly, to determine whether EF training could promote children’s emotional comprehension, we performed 2(group: No-training VS EF training) × 2(test time: pre VS post) repeated measures ANOVA and emotional comprehension was regarded as dependent variable. Results revealed test time the main effect was not significant, *F*(1,49) = 0.104, *p* > 0.05, and group main effect was not significant, *F*(1,49) = 0.630, *p* > 0.05, but the interaction effect between condition and test time was significant, *F*(2,49) = 4.389, *p* < 0.05, η2 = 0.110. Further simple effect analyses showed that EF training group and No-training group were not significant during the pretest, *M*_diff_(I–J) = 0.210, *p* > 0.05, but EF training children during post-test performed was better than No-training group, *M*_diff_(I–J) = 0.721, *P* < 0.001, η2 = 0.093 (see Figure 2-II). The findings suggested that EF training can improve children’s emotional comprehension.

Thirdly, to elucidate whether EF training could predict children’s emotional expression, we performed 2(group: No-training VS EF training) × 2(test time: pre VS post) repeated measures ANOVA and emotional expression was regarded as the dependent variable. The results revealed that test time main effect was not significant, *F*(1,49) = 0.096,*p* > 0.05. The main effect of group was not significant, *F*(1,49) = 0.185,*p* > 0.05, and the interaction between group and test time was significant, *F*(2,49) = 5.170,*p* < 0.01,η2 = 0.127. Further simple effect analyses showed that EF training group and No-training group were not significant during the pretest, *M*_diff_(I–J) = 0.131, *p* > 0.05, and EF training group and No-training group were significant during the post-test, *M*_diff_(I–J) = 0.410, *p* < 0.01, η2 = 0.132 (see Figure 2-III). The findings suggested that EF training can improve children’s emotional expression.Fourthly, in order to examine whether EF training could promote children’s emotional regulation, we performed 2(group: No-training VS EF training) × 2(test time: pre VS post) repeated measures ANOVA. The emotional regulation was the dependent variable. The results revealed that test time main effect was not significant, *F*(1,49) = 0.990, *p* > 0.05. The main effect of group was significant, *F*(1,49) = *F*(1,49) = 3.977, *p* < 0.05, η2 = 0.090, and the interaction between group and test time was significant, *F*(2,49) = 3.495, *p* < 0.05, η2 = 0.127. Further simple effect analyses showed that EF training group and No-training group were not significant during the pretest, *M*_diff_(I–J) = 0.299, *p* > 0.05, and EF training group and No-training group were significant during the post-test, *M*_diff_(I–J) = 0.400, η2 = 0.152 (see Figure 2-IV). The findings suggested that EF training can improve children’s emotional regulation.

### EF Change Predicted EC Change

To further discern the contributions of children’s EC to variance in later EF training effect, correlation and regression analyses were used to achieve this goal. Io order to control the difference of inhibition control during the pretest, we calculated the difference by using post-test minus pretest to represent the development of children’s emotional competence. Firstly, we performed correlation between Δ*EF* and Δ*EC*.

As shown in Table 3, there was a significantly positive correlation between Δ*EF* and Δ*EC*, *r* = 0.493, *p* < 0.01; the relationship between Δ*IC* and Δ*EC* was a significant, *r* = 0.465, *p* < 0.01; the association between Δ*WM* and Δ*EC* was significant, *r* = 0.359, *p* < 0.01, and there was a significant relationship between Δ*PS* and Δ*EC*, *r* = 0.320, *p* < 0.05, but Δ*Flex* was not significantly associated with Δ*EC*, *r* = −0.007, *p* > 0.05. Interestingly, there was a significantly positive relationship between Δ*IC* and Δ*ER*, *r* = 0.498, *p* < 0.01, and the relationship between Δ*WM* and Δ*ECom* was significant, *r* = 0.426 *p* < 0.05. The results suggested that the change of internal sub-capacities (e.g., Δ*IC* and Δ*WM*) could impact the change of emotional competence (e.g., Δ*ER* and Δ*ECom*).

**TABLE 3:** The correlations between the change of EF and EC during pre–posttest.

	**Δ*EF***	**Δ*EC***	**Δ*IC***	**Δ*Flex***	**Δ*WM***	**Δ*PS***	**Δ*ERoc***	**Δ*ECom***	**Δ*EExp***	**Δ*ER***
Δ*EF*	1									
Δ*EC*	0.493**	1								
Δ*IC*	0.716**	0.465**	1							
Δ*Flex*	0.409**	–0.007	0.096	1						
Δ*WM*	0.568**	0.395**	0.324*	–0.150	1					
Δ*PS*	0.73**	0.320*	0.309*	0.149	0.221	1				
Δ*ERoc*	0.266	0.518**	0.348	–0.009	0.091	0.159	1			
Δ*ECom*	0.253	0.606**	–0.047	0.049	0.426*	0.069	0.023	1		
Δ*EExp*	0.033	0.393*	0.168	0.07	0.044	0.193	0.205	0.062	1	
Δ*ER*	0.418*	0.622**	0.488**	0.230	0.148	0.148	0.418*	0.025	0.102	1

*N* = 32. The coefficients of 0.10, 0.30, and 0.50 as small, medium, and large. *p < 0.05; ** < 0.01; *** < 0.001. Δ*EF*: the change of executive function; Δ*IC*: the change of inhibition control; Δ*Flex*: the change of flexibility cognitive; Δ*WM*: the change of working memory; Δ*PS*: the change of problem solving; Δ*EC*: the change of emotional competence; Δ*ERoc*: the change of emotional recognition; Δ*ECom*: the change of emotional comprehension; Δ*EExp*: the change of emotional expression; Δ*ER*: the change of regulation.

To examine EF’s contributions to variance in total emotional competence, we performed the hierarchical linear regression analysis. Regression models include the following two models: (1) first model only included inhibition control and (2) the second model included inhibition control and working memory.

The results showed only inhibition control and working memory were significant predictors compared with all four EF measures. The cognitive flexibility and problem solving cannot be successfully entered into the regression models. These results indicated that only inhibition and working memory can positively predict the development of emotional competence. In summary, these results suggested that the training for children’s EF can improve the development of emotional competence, and both of them could explain 25.3% variation in children’s emotional competence.

To further examine the contributions of the change of inhibition control and working memory to variance in total emotional competence for the No-training group, we also performed the hierarchical linear regression analysis for No-training group. The results showed that children’s change in EF could not predict the change in EC, adjusted *R^2^* = 0.061, *Beta* = 0.218, *t* = 1.190, *p* > 0.05.

## Discussion

Consistent with our hypotheses, the results showed that EF training significantly promoted children’s EC. In terms of pre-and-post-test differences, the results found that (a) EF training group significantly improved the emotional competence (ECs) compared to those of in No-training group. (b) Children’s inhibition control and working memory significantly predicted the development of emotional competence. In our study, we trained children’s EF to improve emotional competence. Training effects were found for emotional comprehension and emotional regulation. Our results suggested that the EF training was able to promote children’s emotional competence.

### EF Training Improved Children’s Emotional Competence

Our results demonstrated that our EF training improved emotional competence in preschool-aged children. Components of EF (e.g., inhibition control) plays an important role in children’s emotional competence. This provides a new evident effect on children’s emotional competence intervention during preschool children. Riggs et al. (2006) found that the better EF performance was always along with the better emotional competence performed. One possible explanation for the finding was that the ability of EF processing needed has some overlapping with emotional competence (Phillips et al., 2003; Chen et al., 2009; Carlson et al., 2013). Traue and Pennebaker (1993) stated that the ability of emotion and inhibition control were significantly correlated. Additionally, children’s effortful control can promote the development of emotional competence (Liew, 2012). Moreover, in clinical studies, inhibition control and working memory played crucial roles in emotional tasks, such as fear extinction (Dillon and Pizzagalli, 2008). Hence, the EF training effect may result from that children’s EF change was used to regulate their emotional competence change, and children’s inhibition control and working memory were the vital factors to the development of emotional competence.

### Inhibition Control

These findings revealed that children’s inhibition control may explain a great amount of variance of children’s emotional competence, especially emotional regulation. Children’s self-control ability could influence the ability of emotional regulation (Carlson and Wang, 2007). In our study, children’s emotional regulation was improved by EF training (e.g., inhibition control). Previous studies have revealed that regulating emotions required ability of inhibition control at the age of 4 to 6. If participants established the link between inhibition and emotional regulation, it would guide them to give better behavioral responses to emotional situations (Denham et al., 2015; Silkenbeumer et al., 2016). It has also been found that participants with better inhibition control ability exhibited better emotional regulation ability compared to lower inhibition control ones (Nakamichi, 2017). And some researchers suggested that participants’ inhibition control could predict their emotional regulation and social competence during preschool (Penela et al., 2015). Our findings were consistent with previous studies. Furthermore, our study provided a new direction of intervening emotional regulation in preschool children by training inhibition control and it would make a contribution to the field from theoretical, methodological, and practically perspective.

### Working Memory

We found that working memory could explain the training effects of EF on emotional competence. There is a considerable evidence for the influence of children’s emotional competence by their working memory (Baddeley, 2013). Neural evidence had proved that the activity in ventral emotional comprehension processing regions was consistent with activity in brain regions related to working memory among PTSD participants (Morey et al., 2009). Another study stated that working memory may explain the frontal lobe involvement in the task processing of emotional comprehension (Mitchell, 2007). Previous research had stated that children’s working memory could influence their emotional comprehension. In this study, the results suggested that training children’s working memory may improve their emotional comprehension, which provides a further evidence to the mental mechanism. The development of working memory had a decisive impact on the development of children’s comprehension between 5 and 11 year-old children (Morra et al., 2011). And a study stated that individual working memory could predict preschoolers’ emotional comprehension performance (Pons et al., 2002; Mutter et al., 2006). In a word, our study can provide new evidence for effectiveness of clinical interventions given that working memory training could improve children’s emotional comprehension.

### Limitations and Future Directions

Our study has some limitations. First, our sample size limited the use of more complex analytical method to detect effect sizes. Thus, recruiting a larger sample may enable researchers to examine these results in the future study. Further longitudinal studies are needed to assess how long the EF training effect can last. Furthermore, the exact neural mechanism will still be needed to investigate in the future by using electroencephalograms (EEG), Functional near-infrared spectroscopy (fNIRS), and other neuroimaging methodologies. Finally, the self-report on daily performance of research assistants will be also needed to collect to examine how the EF training curriculum influences children’s EC as well.

## Conclusion

Our study has revealed that training EF may improve children’s EC. Importantly, children’s inhibition control and working memory could make the change of emotional regulation and emotional comprehension more effectively. These findings in our study can make a theoretical and practical contribution to the field of developmental psychology.

## Data Availability Statement

The datasets generated for this study are available on request to the corresponding author FT.

## Ethics Statement

The studies involving human participants were reviewed and approved by the Ethics Committee of Southwest University. Written informed consent to participate in this study was provided by the participants’ legal guardian/next of kin.

## Author Contributions

QL performed the experiments and wrote the manuscript. PL revised the manuscript. TF and NY provided professional guidance and revised the manuscript.

## Conflict of Interest

The authors declare that the research was conducted in the absence of any commercial or financial relationships that could be construed as a potential conflict of interest.
